# Changes in physiological parameters and thermal comfort when wearing protective clothing in long-range aeromedical evacuation: a prospective, non-blinded, two-stage crossover self-controlled study

**DOI:** 10.1186/s40101-025-00411-9

**Published:** 2025-11-07

**Authors:** Yadi Zhang, Fan Jiang, Zhenyao Song, Jintao Lian, Licun Han

**Affiliations:** 1 Department of Disease Control and Prevention, Air Force Medical Center, Air Force Medical University, PLA, 30# Fucheng Road, Haidian District, Beijing, 100142 China; 2 Section of Health, No. 94804 Unit of the Chinese People’s Liberation Army, Shanghai, 200434 China; 3 Air Force Medical Center, Resident Standardization Training Cadet Corps, PLA, 30# Fucheng Road, Haidian District, Beijing, 100142 China

**Keywords:** Protective clothing, Atmospheric pressure, Protection time

## Abstract

**Background:**

The thermos-physiological characteristics of medical personnel wearing protective clothing during prolonged activities under low oxygen pressure (LOP) and normal oxygen pressure (NOP) are crucial.

**Methods:**

The average age of the 24 participants was 22.13 ± 1.849 years, with an average height of 168.58 ± 6.268 cm, an average weight of 61.62 ± 8.128 kg, and an average BMI of 21.59 ± 1.761 kg/m^2^. Participants were first exposed to an LOP environment. The 6-h experiment involved a three-phase cycle (sitting, walking, and cardiopulmonary resuscitation (CPR)) repeated every hour. After a 2-week washout period, 24 participants were exposed to a NOP environment and repeated the aforementioned experimental procedure. Logistic regression and Cox analysis were used to assess the relationship between different oxygen pressures and human indicators. Restricted cubic spline (RCS) analysis was employed to examine the temporal changes in physiological indicators, and the Kaplan–Meier (K-M) method was used to plot survival curves.

**Results:**

Each observation time point identified 120 min as the optimal protection time, with the greatest intergroup differences observed for both continuous (5/8 variables) and categorical (8/12 variables) parameters at this time point. Stepwise Regression analyses combining logistic and Cox regression identified six significant variables (*P* < 0.05): temperature, SpO₂, pulse pressure, thermal sensation vote (TSV), sultriness, and rating of perceived exertion (RPE). K-M analysis revealed significantly higher probabilities of adverse outcomes in the LOP group compared to the NOP group: SpO₂ abnormalities (HR = 1.439, 95% CI: 1.026–2.017; log-rank *P* = 0.022), High TSV scores (HR = 2.463 [1.537–3.946]; *P* < 0.001), High sultriness scores (HR = 1.603 [1.260–2.040]; *P* < 0.001). RCS analysis of LOP group data showed significant temporal effects: RPE exhibited a nonlinear upward trend (overall *P* < 0.001; nonlinear *P* = 0.002), reaching an inflection point at 200 min. SpO₂ demonstrated linear decline (*P* = 0.002/0.143; inflection point = 200 min). Pulse pressure showed covariate-dependent effects: nonsignificant before adjustment (*P* = 0.430) but significant after adjustment (*P* = 0.008/0.891; inflection point = 200 min).

**Conclusions:**

Our research shows that 120 ~ 200 min is an optimal working time that does not affect the work efficiency of medical personnel.

**Supplementary Information:**

The online version contains supplementary material available at 10.1186/s40101-025-00411-9.

## Introduction

Aeromedical evacuation serves as a critical element in tactical casualty care, employing both fixed-wing aircraft and rotary-wing assets for long-distance patient transport (> 300 km) with continuous in-flight medical management [1]. This transportation modality offers distinct advantages in terms of speed, operational range, and terrain independence, effectively reducing evacuation timelines while maintaining therapeutic continuity. Such capabilities substantially improve casualty survival rates, representing an indispensable component of modern combat medical operations in information warfare scenarios [2]. The European Network for Infectious Diseases categorizes highly infectious diseases as those demonstrating human-to-human transmission potential, life-threatening severity, and necessitating specialized containment protocols [3]. During the COVID-19 pandemic, Western nations have implemented aeromedical evacuation systems for infectious case management [4–6]. Current protocols mandate comprehensive personal protective equipment (PPE), including N95 respirators, impermeable gowns, and medical gloves, for all patient contacts. Long-range aeromedical evacuation (operationally defined as > 4 h in Chinese aviation standards [7]) present unique physiological challenges when conducted in military transport aircraft cabin environments. Medical personnel wearing non-breathable PPE under hypobaric conditions experience compounded thermal stress, with documented adverse effects on both physiological parameters and cognitive performance [8]. Therefore, studying the changes in physiological indicators of medical personnel under low-pressure conditions over time and their impact on human fatigue is of great significance for safely completing tasks.

Extensive research has been conducted on human thermal comfort in hot and humid environments, with most studies focusing on either the effects of protective clothing materials or isolated environmental stressors (elevated temperature, humidity, or hypobaric conditions). Medical personal protective equipment (PPE) induces heat fatigue and thermal discomfort, ultimately reducing work tolerance time and impairing both physical and cognitive performance [9–11]. Troynikov et al. systematically evaluated protective garment functionality through material analysis and thermal manikin testing, establishing key relationships between fabric properties and thermos physiological comfort [12]. Potter et al. quantified heat stress risks from impermeable PPE in tropical environments, developing core temperature-based models to predict safe work durations during the West African Ebola outbreak [13]. Research has demonstrated that high temperatures can lead to a gradual increase in respiratory rate and subjective symptoms in the human body, with significant rises in core temperature and heart rate [14, 15]. Additionally, the incidence of cardiovascular diseases is increased [16, 17]. On the other hand, for individuals who are exposed to low-pressure environments in the short term, the reduction in oxygen partial pressure (hypobaric hypoxia) is considered the main cause affecting their health condition [18–20]. Studies have shown that with increasing altitude, human heart rate increases linearly, while SpO₂ and work capacity decrease, and left ventricular mechanics are altered [21–23]. While Xue [24] and Zhou [25] examined combined heat-hypobaric stress in aviation contexts, no studies have investigated the synergistic effects of prolonged PPE use under low-pressure conditions. This represents a critical knowledge gap regarding aeromedical personnel's physiological adaptations and operational limits during extended medical evacuations.

Our study aims to simulate the process of long-range aeromedical evacuation of infectious disease patients, observing the physiological indicators and subjective feelings of medical personnel involved in the mission while they are in a secondary protective state. During the transport, medical personnel spend most of their time sitting still or periodically checking on patients, adhering to the principle of prioritizing patient transport over treatment [26]. Only when patients show critical symptoms do medical personnel choose appropriate emergency measures to resuscitate them. Cardiopulmonary resuscitation (CPR) is the simplest, most commonly used, most physically demanding, equipment-free, highly standardized, and easily scalable emergency measure. It is a representative medical intervention that is easy to train and implement, and selecting a physically demanding measure (since CPR requires five cycles, the physical exertion is extremely significant) makes it easier to observe the participant's tolerance and endurance time under low-pressure conditions, resulting in more rigorous and accurate experimental results regarding the optimal protective time of protective clothing in LOP/NOP environments. Therefore, this study analyzed the effects of low air pressure and protection time on human fatigue by monitoring the change patterns of physiological indicators of 24 participant wearing protective gear in normal air pressure and low-pressure oxygen chambers to do specific activities for six hours and filling in subjective questionnaires at specific stages, to provide data support for the control of the length of personal protection of healthcare workers during the airlift evacuation of patients with infectious diseases.

## Materials and methods

### Participants

Inclusion Criteria: 1) Height: Female (160–165 cm), Male (172–178 cm), 2) BMI: 18.5–24.5 kg/m^2^. Exclusion Criteria: 1) Heart rate (HR): 60–100 times/min, 2) Systolic blood pressure (SBP): 90–120 mmHg, Diastolic pressure (DP): 60–90 mmHg, 3) Respiratory rate: 16–20 times/min, 4) SpO_2_: 95–100%, 5) Temperature: 36.1–37.2℃, 6) recent eye surgery, fixed jaw surgery, abdominal surgery, 7) recent acute gastrointestinal illness and severe colds, 8) airsickness.

**Fig. 1:**
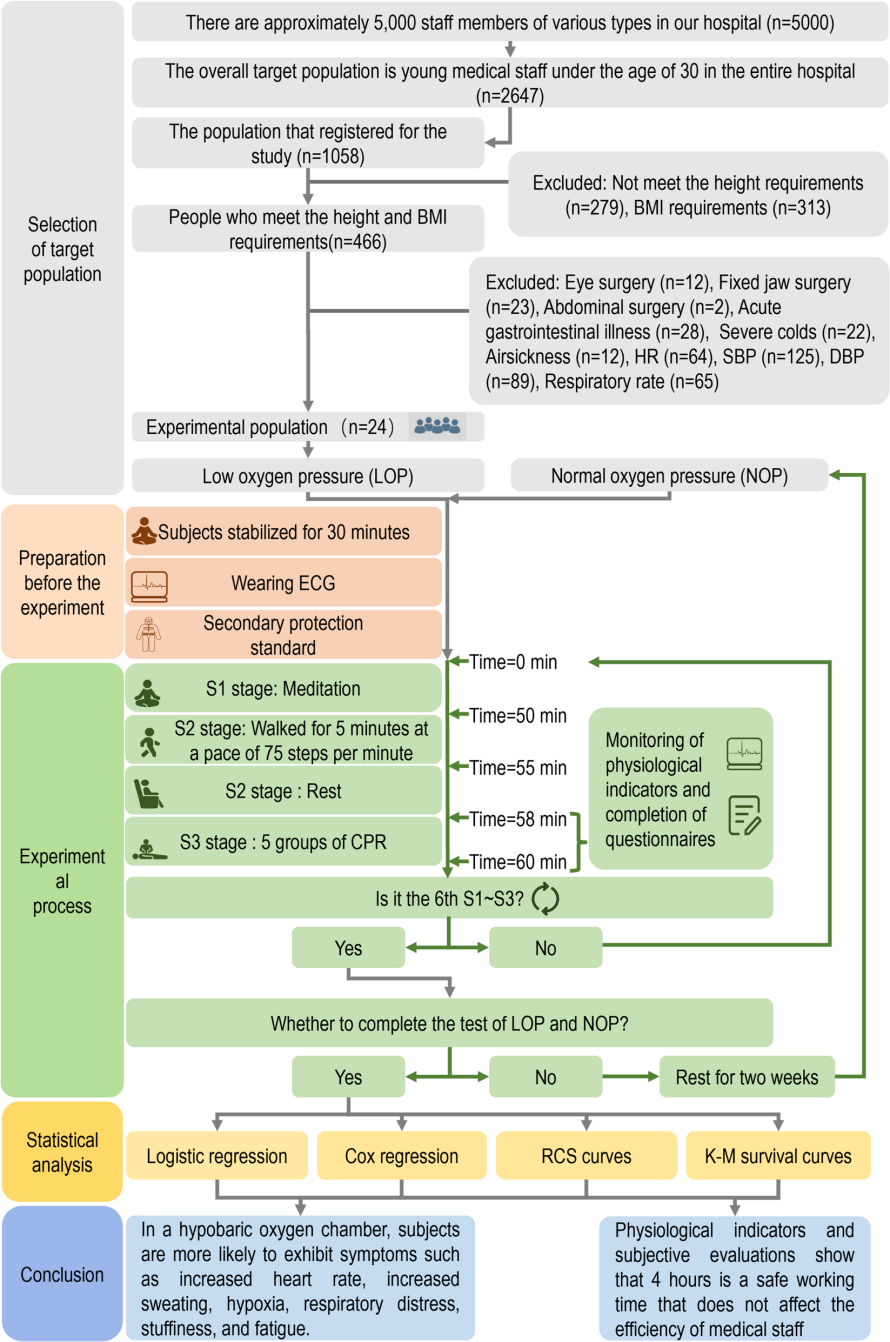
Flowchart for monitoring physiological indicators and subjective perception analysis of 24 participant in normal pressure and low-pressure oxygen chambers. The process includes the preparation stage before the experiment (setting of experimental site parameters, wearing of protective equipment and monitoring devices by participant, etc.) and the formal experiment. The formal experiment consists of three stages, namely, the participant sitting quietly for 50 min (S1 stage), walking for 5 min at a pace of 75 steps per minute and resting for 3 min (S2 stage), and performing 1 set of CPR (S3 stage). After stepping and CPR, the participant filled out a subjective questionnaire for 6 h, repeating stages S1 to S3 every hour. The participant first completed the experiment in a low-pressure oxygen chamber, rested for two weeks, and then completed the human thermal physiology experiment under normal pressure environment

Our tertiary hospital employs approximately 5,000 healthcare professionals across multiple disciplines, including clinical staff (physicians, nurses), allied health professionals (technicians, pharmacists), trainees (interns, residents, postgraduate students), and support personnel (field workers, cleaners, administrators). The study targeted medical staff aged < 30 years (*n* = 2,674), from which 1,058 volunteers initially enrolled. Preliminary screening based on anthropometric parameters (height, BMI) yielded 466 eligible candidates. The application of exclusion criteria reduced the pool to 24 participants. Standardized physiological measurements were conducted using identical electrocardiograph monitors (same brand/model) to assess SpO₂, Blood pressure (systolic/diastolic), Heart rate, and Respiratory rate. Body temperature was measured using calibrated thermometers. Participants were selected through randomized sampling without preferential selection. The complete screening protocol is illustrated in Fig. 1.

The "China National Physical Fitness Survey Report" shows that the average height of males aged 18–25 is 172.4 cm, while the average height of females is approximately 160 cm [27]. Taller individuals exhibit greater body surface area (BSA), which increases resting energy expenditure due to enhanced thermoregulatory heat loss [28]. Height standardization minimizes baseline physiological variability, as cardiovascular demands scale with stature—taller individuals demonstrate elevated cardiac positioning and require greater blood volume and cardiac output to maintain homeostasis [29]. When participants are wearing protective clothing and in a state of hypoxia, height can indirectly affect physiological indicators such as core body temperature, heart rate, respiratory rate, and fatigue level. In addition, another important reason is that the commonly available sizes are only S, M, and L, which are suitable for individuals between 160 and 185 cm in height [30]. Moreover, since protective clothing usually requires an inner layer, most infectious disease protection processes involve choosing a size larger than the individual's actual size. This can create practical difficulties in protection for taller individuals (for example, those over 190 cm). Therefore, ensuring that the participants' height falls within a certain range can control the physiological differences derived from body size and surface area, reducing research errors caused by height-related inherent factors such as circulatory metabolism. The formula for calculating BMI (kg/m^2^) is weight (kg) divided by the square of height (m). Pulse pressure (mmHg) is a derived indicator of blood pressure, referring to the difference between systolic blood pressure and diastolic blood pressure. It mainly reflects the elasticity of the aorta. In a low-pressure environment, pulse pressure is more sensitive than blood pressure and has higher predictive value [31]. Monitoring changes in pulse pressure dynamically can more comprehensively reflect and assess the impact of low-pressure environments on participants' cardiac function and help prevent the occurrence and development of adverse events. To facilitate analysis and better interpret the results, the following derived variables are defined: SpO_2__normal/abnormal, temperature_normal/abnormal, respiratory rate_normal/abnormal, heart rate_normal/abnormal, diastolic pressure_normal/abnormal, and pulse pressure_normal/abnormal. These variables indicate whether the values of SpO_2_, body temperature, respiratory rate, heart rate, diastolic blood pressure, and pulse pressure are within the normal range, thereby providing an objective and comprehensive assessment of the impact of low-pressure environments on the human body.

Twenty-four participants were ultimately enrolled in the trial, consisting of 12 males and 12 females. Participant were instructed to maintain their normal daily routines and to avoid strenuous exercise within 24 h before the experiment. Additionally, they were required to familiarize themselves with the experimental content and precautions to ensure that their actions did not adversely affect the results.

### Design of experiments

Prior to testing, participants underwent a 15-min rest period outside the experimental site to ensure bowel evacuation and physiological stabilization. The low-pressure oxygen chamber was maintained at 29.5 °C with 53.7% relative humidity and 571 m^3^/h airflow. The chamber simulated an altitude of 2500 m, corresponding to an atmospheric pressure of 748.50 hectopascals based on standard altitude-pressure conversion formulae. Participants entered the LOP environment wearing electrocardiogram (ECG) monitors in compliance with secondary protection protocols [32]. Medical disposable protective suits (Model II one-piece with foot covers, batch 2,022,110,801; Shandong Angyang Medical Technology Co., Ltd.) were utilized, with all units within their 3-year validity period. Participants sat still for 50 min and then walked for 5 min at a pace of 75 steps per minute, rested for 3 min, and subsequently performed one consecutive set of CPR (CPR requires five cycles, the physical exertion is extremely significant) on a simulator. Fill out the questionnaire after completing CPR (Supplementary Material 1). The trial lasted for 6 h, with the three specific activities—sitting, walking, and CPR—repeated every hour. The normal pressure control experiment was conducted after a two-week washout period, during which participants were confirmed free from acute/chronic illnesses, infections, or confounding exposures (e.g., hyperthermia, medications, or strenuous exercise). All participants maintained normal resting physiological parameters throughout. The normal pressure environment replicated the LOP conditions (29.5 °C, 53.5% humidity, 571 m^3^/h airflow). Prior to experimentation, all 24 participants received standardized CPR training and demonstrated proficiency in meeting compression depth (5–6 cm) and rate (100–120 compressions/minute) guidelines. Two senior nursing instructors supervised all experimental sessions to ensure procedural adherence.

This study employed multiple validated scales to assess participants' subjective perceptions. Thermal Comfort Vote (TCV) measured comfort levels using the 7-point ASHRAE scale, and Thermal Acceptance Vote (TAV) assessed acceptability via the 4-point ISO 10551 scale with reference to Zhao et al. [33]. The sultriness indicator is evaluated referring to the study by Cui Xinwen [34]. Rating of Perceived Exertion (RPE) refers to the subjective perception of fatigue. In this study, we used the Borg scale to assess the participants' subjective level of fatigue [35]. Human fatigue levels range from 6 to 20, with higher numbers indicating greater fatigue. The assessment of the two indicators, thermal sensation and humidity sensation, referred to the study by Zhao Yingying [33] and the 10-point scale of ISO 10551 for evaluation. The thermal comfort indicator is evaluated using the 7-point scale of ASHRAE.

To determine whether the SpO_2_ level is within the normal range, we referred to the research of S Porcelli [36]and J Canet [37]. It is considered abnormal if the SpO_2_ level is below 90% in low-pressure environments and below 95% in normal-pressure environments. Informed consent was obtained and signed by the study participants (Supplementary Material 2), and the study was ethically reviewed by the Ethics Committee of the Chinese Air Force Specialty Medical Center. Grant number: Air Force Specialty Medical Center (Research) No. 2023–60-PJ01.

### Statistical analysis

Data were statistically analyzed using the autoReg package in R Studio to generate a baseline questionnaire and perform Cox and logistic analyses. We employed univariate logistic regression and Cox regression. Variables with *P*-values less than 0.05 were included in the multivariate analysis. Variables in the multivariate analysis were further screened using backward stepwise regression to identify the optimal variables. Variables included through backward stepwise regression were considered the best variables, and these variables underwent VIF (Variance Inflation Factor) testing, with variables having a VIF > 10 being excluded. Using the Storm Statistics online server [38], we plotted RCS curves to explore the relationships between SpO₂, temperature, RPE, pulse pressure, and protection time (continuous independent variables), with the number of nodes automatically selected based on AIC. We also adjusted for confounding factors: TSV, heat stress, and both. Log-rank tests and K-M survival analysis were used to compare differences between the two groups in terms of TSV, SpO2, and heat stress. To more intuitively demonstrate the differences between LOP and NOP at different time points, statistical tests were conducted separately for binary and continuous variables using the gtsummary R package on data from different time points (1, 2, 3, 4, 5, and 6 h). Visualization was performed using ggplot, with continuous variables analyzed using the Wilcoxon test and categorical variables using the Fisher test. The time trend of continuous variables is used with the geom_smooth function, method = "loess", span = 0.3, se = TRUE. *P*-values < 0.001 are marked as ***, *P*-values < 0.01 are marked as **, and *P*-values < 0.05 are marked as *. A *p*-value < 0.05 was defined as statistically significant.

## Results

### Baseline characteristics

This prospective, observational, non-blinded, two-stage crossover study involved all 24 participants completing both experimental conditions: NOP and LOP environments. Participants first underwent the LOP protocol while wearing standardized protective clothing and continuous electrocardiographic monitoring, adhering to the experimental procedure detailed in Fig. 1. Following a mandatory two-week washout period—during which participants maintained normal activities while confirming the absence of acute/chronic illnesses, infectious diseases, or confounding exposures (e.g., hyperthermia, medication use, intense exercise, or significant psychological stress)—the same cohort repeated the protocol under NOP conditions. All participants demonstrated normal resting physiological parameters throughout the study period. The sole experimental manipulation involved environmental pressure variation between conditions, ensuring identical baseline characteristics across both experimental groups through this self-controlled design.

The baseline characteristics of the participants in this study are as follows: the mean age of the participants was 22.13 ± 1.849 years, the mean height was 168.58 ± 6.268 cm, the mean weight was 61.62 ± 8.128 kg, and the mean BMI was 21.59 ± 1.761 kg/cm^2^. The results showed that the temperature (*P* < 0.001), SpO_2_ (*P* < 0.001), respiration rate (*P* = 0.003), SBP (*P* = 0.001), and pulse pressure (P < 0.001) of participants in the LOP group were significantly lower than those in the NOP group. In contrast, the HR (*P* < 0.001) and DP (*P* < 0.001) were significantly higher in the LOP group compared to the NOP group. The subjective perception survey showed that the TCV (*P* < 0.001) of participants in the LOP group was lower than those in the NOP group. Conversely, the TSV(*P* < 0.001), Humid Sensation Vote (HSV) (*P* < 0.001), RPE (*P* < 0.001), Sultriness (*P* < 0.001), and Dizziness (*P* < 0.001) scores were higher in the LOP group, which indicated that participant were more prone to profuse sweating, fatigue, stifling, and dizziness in the LOP (Table 1).

**Table 1:** Baseline characteristics of all study Populations

Group	NOP (*N* = 288)	LOP (*N* = 288)	Total	*p*	sig	class	ptest
Time_min	207.50 [117.50;297.50]	207.50 [117.50;297.50]	207.50 [117.50;297.50]	*p* = 1.000		continuous	non-normal
Thermal.sensation				*p* < 0.001	***	categorical	Pearson's Chi-squared test
- 0	98 (34.03%)	3 (1.04%)	101 (17.53%)				
- 1	70 (24.31%)	23 (7.99%)	93 (16.15%)				
- 2	50 (17.36%)	42 (14.58%)	92 (15.97%)				
- 3	70 (24.31%)	191 (66.32%)	261 (45.31%)				
- 4	0 (0.0%)	29 (10.07%)	29 (5.03%)				
Humid.sensation						categorical	
- −1	3 (1.04%)	0 (0.0%)	3 (0.52%)				
- 0	117 (40.62%)	2 (0.69%)	119 (20.66%)				
- 1	96 (33.33%)	75 (26.04%)	171 (29.69%)				
- 2	47 (16.32%)	82 (28.47%)	129 (22.40%)				
- 3	25 (8.68%)	120 (41.67%)	145 (25.17%)				
- 4	0 (0.0%)	9 (3.12%)	9 (1.56%)				
Sultriness				*p* < 0.001	***	categorical	Pearson's Chi-squared test
- 0	77 (26.74%)	6 (2.08%)	83 (14.41%)				
- 1	154 (53.47%)	116 (40.28%)	270 (46.88%)				
- 2	35 (12.15%)	99 (34.38%)	134 (23.26%)				
- 3	10 (3.47%)	56 (19.44%)	66 (11.46%)				
- 4	12 (4.17%)	11 (3.82%)	23 (3.99%)				
Dizziness				p < 0.001	***	categorical	Fisher's Exact Test for Count Data
- 0	263 (91.32%)	209 (72.57%)	472 (81.94%)				
- 1	24 (8.33%)	59 (20.49%)	83 (14.41%)				
- 2	1 (0.35%)	18 (6.25%)	19 (3.30%)				
- 3	0 (0.0%)	2 (0.69%)	2 (0.35%)				
Thermal.comfort				*p* < 0.001	***	categorical	Fisher's Exact Test for Count Data
- −2	33 (11.46%)	2 (0.69%)	35 (6.08%)				
- −1	93 (32.29%)	179 (62.15%)	272 (47.22%)				
- 0	157 (54.51%)	102 (35.42%)	259 (44.97%)				
- 1	5 (1.74%)	3 (1.04%)	8 (1.39%)				
- 2	0 (0.0%)	2 (0.69%)	2 (0.35%)				
Thermal.accept						categorical	
- −3	0 (0.0%)	14 (4.86%)	14 (2.43%)				
- −2	0 (0.0%)	141 (48.96%)	141 (24.48%)				
- −1	82 (28.47%)	113 (39.24%)	195 (33.85%)				
- 0	193 (67.01%)	20 (6.94%)	213 (36.98%)				
- 1	10 (3.47%)	0 (0.0%)	10 (1.74%)				
- 2	3 (1.04%)	0 (0.0%)	3 (0.52%)				
Human.fatigue	10.00 [8.00;12.00]	12.00 [11.00;13.00]	11.00 [10.00;13.00]	*p* < 0.001	***	continuous	non-normal
Temperature	36.70 [36.50;36.90]	36.50 [36.10;36.80]	36.60 [36.30;36.90]	*p* < 0.001	***	continuous	non-normal
SpO_2_	97.00 [95.00;98.00]	90.00 [89.00;92.00]	93.00 [90.00;97.00]	*p* < 0.001	***	continuous	non-normal
Respiratory.rate	26.00 [22.00;29.00]	24.00 [20.00;28.00]	25.00 [21.00;29.00]	*p* = 0.003	**	continuous	non-normal
Heart.rate	104.00 [95.00;114.00]	111.00 [101.00;123.00]	107.00 [98.00;118.00]	*p* < 0.001	***	continuous	non-normal
Diastolic.pressure	67.00 [61.00;73.00]	71.00 [65.00;76.00]	69.00 [63.00;74.00]	*p* < 0.001	***	continuous	non-normal
Systolic.blood.pressure	115.20 ± 11.64	112.01 ± 10.29	113.61 ± 11.09	*p* = 0.001	***	continuous	normal
Pulse.pressure	46.00 [40.00;53.00]	41.00 [35.00;47.00]	44.00 [37.00;51.00]	*p* < 0.001	***	continuous	non-normal

*NOP* Normal oxygen pressure, *LOP* Low oxygen pressure

### Logistic regression analysis

Perform univariate logistic regression analysis on the six physiological indicators that were monitored, and identify statistically significant features with P < 0.05 in the univariate analysis. All variables in the univariate analysis had p-values less than 0.05 and were therefore included in the multivariate analysis. Stepwise backward regression did not eliminate any non-significant variables. Consequently, the variables retained in the univariate, multivariate, and stepwise regression analyses were SpO₂, human fatigue, temperature, respiratory rate, pulse pressure, thermal sensation, and thermal comfort. The results showed that temperature (OR = 0.22, 95% CI 0.14–0.36), SpO_2_ (OR = 0.61, 95% CI 0.57–0.66), Respiration rate (OR = 0.97, 95% CI 0.95–0.99), and pulse pressure (OR = 0.95, 95% CI 0.93–0.96) were significantly lower in participant in the LOP group compared to those in the NOP group, while DP (OR = 1.04, 95% CI 1.02–1.06) were higher than those in participant in the NOP group (Table 1). Multifactorial logistic regression analysis of all significant factors showed that temperature (OR = 0.18, 95% CI 0.06–0.52), SpO_2_ (OR = 0.63, 95% CI 0.57–0.7), TSV score value 1 (OR = 14.59, 95% CI 3.08–69.03), TSV score value 2 (OR = 36.01, 95% CI 7.85–165.08), TSV score value 3 (OR = 225.87, 95% CI 45.41–1123.50) were significantly different between the two groups (Table 2). Comparative analysis revealed significant intergroup differences in physiological parameters, with the LOP group demonstrating particularly marked alterations. Participants in the LOP condition exhibited elevated heart rates and reduced peripheral SpO₂ levels, consistent with hypoxic physiological stress. These findings suggest that hypobaric environments exert more pronounced adverse effects on human physiology compared to normobaric conditions when controlling for equivalent protective measures and physical activity levels.

**Table 2:** Logistic regression of two groups of participants

Dependent: Group		OR (univariable)	OR (multivariable)	OR (final)
SpO_2_	continuous	0.61 (0.57–0.66, *p* <.001)	0.63 (0.57–0.70, *p* <.001)	0.63 (0.57–0.70, *p* <.001)
Diastolic.pressure	continuous	1.04 (1.02–1.06, *p* <.001)	1.04 (0.99–1.09, *p* =.090)	1.04 (0.99–1.09, *p* =.090)
Human.fatigue	continuous	1.36 (1.25–1.48, *p* <.001)	1.58 (1.31–1.91, *p* <.001)	1.58 (1.31–1.91, *p* <.001)
Temperature	continuous	0.22 (0.14–0.36, *p* <.001)	0.18 (0.07–0.47, *p* <.001)	0.18 (0.07–0.47, *p* <.001)
Respiratory.rate	continuous	0.97 (0.95–0.99, *p* =.002)	0.96 (0.92–1.00, *p* =.053)	0.96 (0.92–1.00, *p* =.053)
Pulse.pressure	continuous	0.95 (0.93–0.96, *p* <.001)	0.93 (0.90–0.97, *p* <.001)	0.93 (0.90–0.97, *p* <.001)
Thermal.sensation	0			
	1	10.73 (3.10–37.15, *p* <.001)	14.59 (3.08–69.03, *p* <.001)	14.59 (3.08–69.03, *p* <.001)
	2	27.44 (8.10–92.93, *p* <.001)	36.01 (7.85–165.08, *p* <.001)	36.01 (7.85–165.08, *p* <.001)
	3	89.13 (27.36–290.35, *p* <.001)	225.87 (45.41–1123.50, *p* <.001)	225.87 (45.41–1123.50, *p* <.001)
	4	1,389,797,202.56 (0.00-Inf, *p* =.977)	259,232,261,043,251,136.00 (0.00-Inf, *p* =.983)	259,232,261,043,251,136.00 (0.00-Inf, *p* =.983)
Thermal.comfort	−2			
	−1	31.76 (7.46–135.27, *p* <.001)	2,062,613,656.06 (0.00-Inf, *p* =.984)	2,062,613,656.06 (0.00-Inf, *p* =.984)
	0	10.72 (2.52–45.65, *p* =.001)	7,448,266,416.84 (0.00-Inf, *p* =.983)	7,448,266,416.84 (0.00-Inf, *p* =.983)
	1	9.90 (1.31–74.73, *p* =.026)	1,781,747,163.80 (0.00-Inf, *p* =.984)	1,781,747,163.80 (0.00-Inf, *p* =.984)
	2	34,949,969.63 (0.00-Inf, *p* =.978)	2,203,598,171,625,637,632.00 (0.00-Inf, *p* =.995)	2,203,598,171,625,637,632.00 (0.00-Inf, *p* =.995)

The OR (final) is the result of stepwise backward logistic regression

### Cox regression analysis

The inclusion of physiological indicators and subjective feelings in Cox regression analysis revealed that temperature (HR = 0.50, 95% CI 0.37–0.69, *P* < 0.001), SpO_2_ (HR = 0.92, 95% CI 0.91–0.94, *P* < 0.001), respiration rate (HR = 0.96, 95% CI 0.95–0.98, *P* < 0.001), SBP (HR = 0.98, 95% CI 0.97–0.99, *P* < 0.001), pulse pressure (HR = 0.97, 95% CI 0.96–0.98, *P* < 0.001), and TSV score value 1 (HR = 9.13, 95% CI 2.74- 30.40, *P* < 0.001), 2 (HR = 19.90, 95% CI 6.17–64.23, *P* < 0.001), 3 (HR = 27.28, 95% CI 8.72–85.38, P < 0.001), 4 (HR = 25.42, 95% CI 7.74- 83.56, *P* < 0.001), as well as Sultriness score value 1 (HR = 5.09, 95% CI 2.24–11.56, *P* < 0.001), 2 (HR = 8.04, 95% CI 3.53–18.34, *P* < 0.001), 3 (HR = 8.49, 95% CI 3.66–19.71, *P* < 0.001), 4 (HR = 3.92, 95% CI 1.45–10.61, *P* = 0.01), TCV score value −1 (HR = 13.64, 95% CI 3.38–54.95, P < 0.001), and RPE (HR = 1.08, 95% CI 1.03–1.14, *P* < 0.001) were statistically significantly different (Table 3). All variables that were significant in the univariate Cox regression analysis were included in the multivariate regression analysis. This analysis showed that, compared to the NOP group, participants in the LOP group were 0.97 times more likely to experience a decrease in SpO_2_ (HR = 0.97, 95%CI 0.95–0.99) and 0.99 times more likely to have a reduction in pulse pressure (HR = 0.99, 95%CI 0.97- 1.00). The probability of selecting TSV score value 4 (HR = 33.51, 95%CI 8.68–129.41) was 33.51 times more likely than in the NOP group, and the likelihood of an increase in RPE (HR = 1.11, 95%CI 1.02–1.20) was 1.11 times more likely than in the NOP group (Table 3). Elevated scores on the TSV, TCV, and sultriness perception scales were associated with heightened thermal discomfort among participants. Subjective fatigue levels, as measured by the RPE, demonstrated a positive correlation with reported fatigue intensity. Comparative analysis revealed significant between-group differences in these subjective measures, indicating that hypobaric conditions were more likely to induce physiological stress responses, including hypoxia, tachycardia, dyspnea, and fatigue, when compared to normobaric environments.

**Table 3:** Cox analysis of two groups of participants

Dependent: Surv(Time_min, Group)		HR (univariable)	HR (multivariable)	HR (final)
SpO_2_	continuous	0.92 (0.91–0.94, *p* <.001)	0.95 (0.92–0.99, *p* =.008)	0.97 (0.95–0.99, *p* =.008)
Systolic.blood.pressure	continuous	0.98 (0.97–0.99, *p* <.001)	0.98 (0.96–1.00, *p* =.101)	1.00 (0.98–1.01, *p* =.939)
Human.fatigue	continuous	1.08 (1.03–1.14, *p* <.001)	1.00 (0.86–1.17, *p* =.985)	1.11 (1.02–1.20, *p* =.010)
Temperature	continuous	0.50 (0.37–0.69, *p* <.001)	0.81 (0.44–1.49, *p* =.499)	0.66 (0.46–0.95, *p* =.025)
Respiratory.rate	continuous	0.96 (0.95–0.98, *p* <.001)	0.96 (0.94–0.99, *p* =.002)	0.97 (0.95–0.99, *p* =.003)
Pulse.pressure	continuous	0.97 (0.96–0.98, *p* <.001)	0.99 (0.97–1.01, *p* =.336)	0.99 (0.97–1.00, *p* =.176)
Thermal.sensation	0			
	1	9.13 (2.74–30.40, *p* <.001)	9.04 (2.49–32.80, *p* =.001)	11.12 (3.08–40.09, p <.001)
	2	19.90 (6.17–64.23, *p* <.001)	57.54 (14.64–226.11, *p* <.001)	23.34 (6.60–82.55, p <.001)
	3	27.28 (8.72–85.38, *p* <.001)	70.54 (17.59–282.91, *p* <.001)	30.38 (8.51–108.39, p <.001)
	4	25.42 (7.74–83.56, *p* <.001)	53.05 (11.72–240.12, *p* <.001)	33.51 (8.68–129.41, p <.001)
Sultriness	0			
	1	5.09 (2.24–11.56, *p* <.001)	0.77 (0.28–2.09, *p* =.604)	0.79 (0.32–1.96, *p* =.604)
	2	8.04 (3.53–18.34, *p* <.001)	0.61 (0.20–1.85, *p* =.378)	0.66 (0.24–1.78, *p* =.407)
	3	8.49 (3.66–19.71, *p* <.001)	0.44 (0.13–1.51, *p* =.191)	0.58 (0.20–1.68, *p* =.316)
	4	3.92 (1.45–10.61, *p* =.007)	0.37 (0.08–1.64, *p* =.192)	0.47 (0.14–1.64, *p* =.238)
Thermal.comfort	−2			
	−1	13.64 (3.38–54.95, *p* <.001)	9.83 (2.24–43.19, *p* =.002)	10.49 (2.52–43.69, *p* =.001)
	0	8.41 (2.08–34.10, *p* =.003)	13.38 (2.90–61.82, *p* =.001)	14.21 (3.34–60.50, *p* <.001)
	1	7.02 (1.17–42.06, *p* =.033)	13.77 (1.98–95.85, *p* =.008)	14.71 (2.35–92.26, *p* =.004)
	2	248.46 (34.14–1808.18, *p* <.001)	41,651,953,397.19 (0.00-Inf, *p* =.998)	321.62 (41.73–2479.08, *p* <.001)

The HR (final) is the result of stepwise backward Cox regression

### RCS curve

**Fig. 2:**
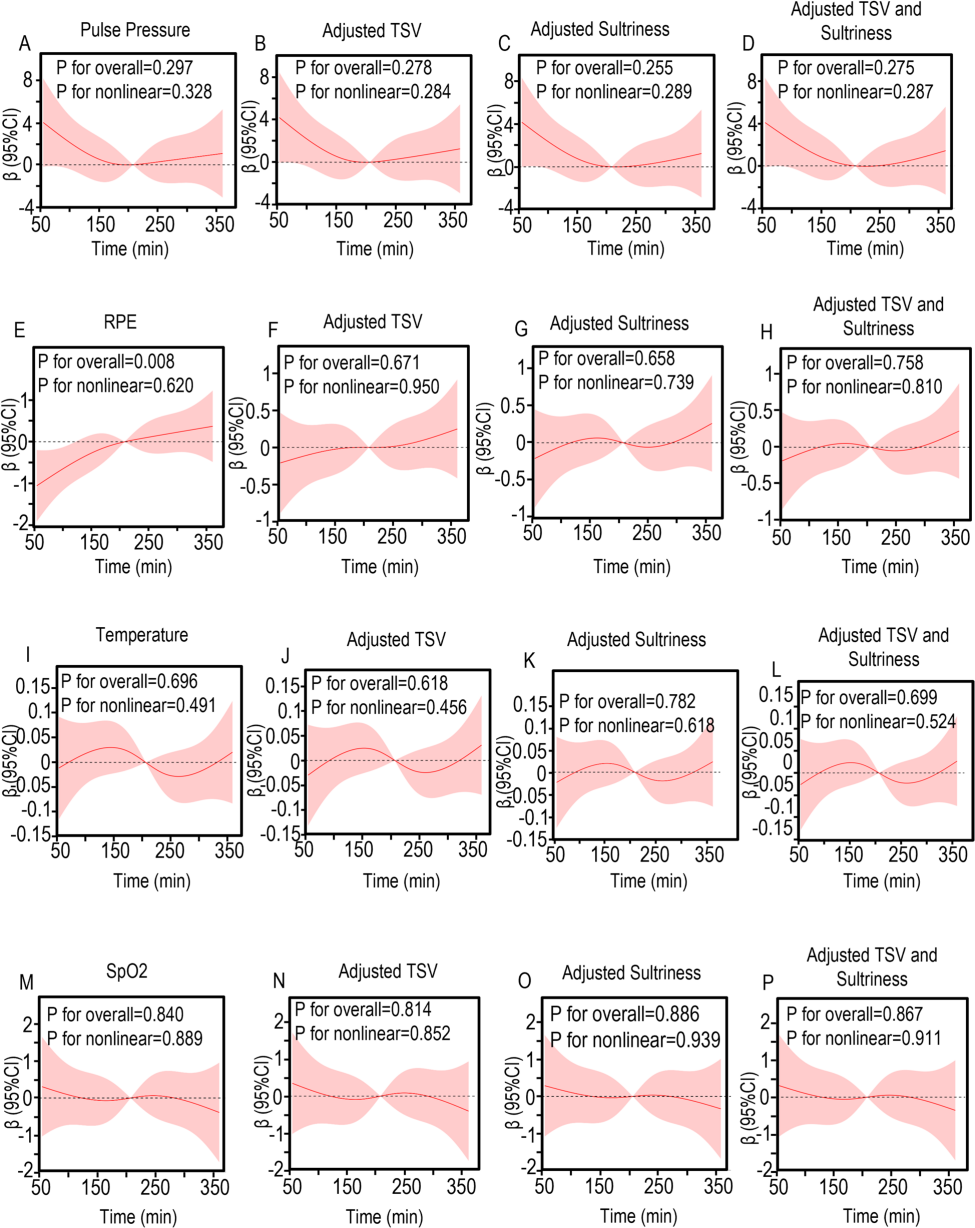
Nonlinear relationship between pulse pressure, RPE, temperature, SpO_2_, and protection time in NOP group. The relationship was evaluated by RCS after adjustment for TSV, Sultriness, and both. The solid lines in the figure represents predicted values of nonlinear models, and the shaded regions represents the 95% CI. **A** Pulse pressure; **B** Pulse pressure after adjusting TSV; **C** Pulse pressure after adjusting Sultriness; **D** Pulse pressure after adjusting TSV and Sultriness; **E** RPE; **F** RPE after adjusting TSV; (G) RPE after adjusting Sultriness; (H) RPE after adjusting TSV and Sultriness; **I** Temperature; **J** Temperature after adjusting TSV; **K** Temperature after adjusting Sultriness; **L** Temperature after adjusting TSV and Sultriness; **M** SpO_2_; **N** SpO_2_ after adjusting TSV; **O** SpO_2_ after adjusting Sultriness; **P** SpO_2_ after adjusting TSV and Sultriness; CI, confidence intervals; HR, hazard ratios; RCS, restricted cubic spline

**Fig. 3:**
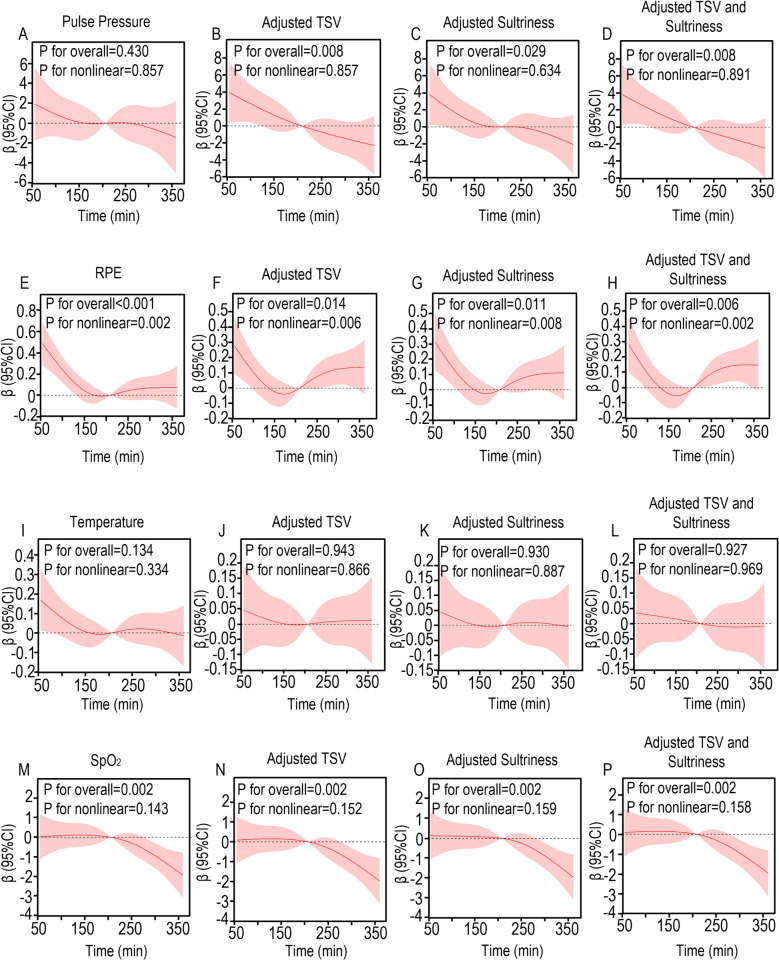
Nonlinear relationship between pulse pressure, RPE, temperature, SpO_2_, and protection time in LOP group. The relationship was evaluated by RCS after adjustment for TSV, Sultriness, and both. The solid lines in the figure represents predicted values of nonlinear models, and the shaded regions represents the 95% CI. **A** Pulse pressure; **B** Pulse pressure after adjusting TSV; **C** Pulse pressure after adjusting Sultriness; **D** Pulse pressure after adjusting TSV and Sultriness; **E** RPE; **F** RPE after adjusting TSV; **G** RPE after adjusting Sultriness; **H** RPE after adjusting TSV and Sultriness; **I** Temperature; **J** Temperature after adjusting TSV; **K** Temperature after adjusting Sultriness; **L** Temperature after adjusting TSV and Sultriness; **M** SpO_2_; **N** SpO_2_ after adjusting TSV; **O** SpO_2_ after adjusting Sultriness; **P** SpO_2_ after adjusting TSV and Sultriness; CI, confidence intervals; HR, hazard ratios; RCS, restricted cubic spline

The relationship between pulse pressure, RPE, temperature, and SpO_2_ and the duration of protection in participants in the LOP and NOP groups was analyzed using nonlinear trend RCS regression. In the NOP group, regardless of whether covariates were adjusted, pulse pressure (*P* for overall = 0.297), SpO_2_ (P for overall = 0.840), and temperature (*P* for overall = 0.696) did not change significantly with protective time (Fig. 2A ~ 2D/Fig. 2I ~ 2L/Fig. 2M ~ 2P). RPE (P for overall = 0.008) showed a significant linear relationship with protective time before adjusting for covariates, meaning that RPE increased as time prolonged (Fig. 2E). However, after adjusting the variables, the linear relationship disappeared (*P* for overall = 0.758), indicating that RPE is affected by TSV and sultriness (Fig. 2F ~ 2H). In the LOP group, the RPE reached a turning point at 200 min, indicating that, compared to the initial time point of 50 min, at 200 min, the red solid line was on the gray line with β (regression coefficient) = 0, indicating an upward trend (Fig. 3E ~ 3H). The overall P-value is less than 0.001 (*P* for overall < 0.001), which strongly indicates that time has a significant impact on RPE. The non-linear *P*-value of 0.002 (P for nonlinear = 0.002) suggests that the data trend is close to a curve, specifically an "L" shape, with no obvious linear change (Fig. 3E ~ 3H). SpO2 shows a turning point at 200 min, indicating that compared to the initial time point of 50 min, at 200 min, the red solid line is below the grey line representing β (regression coefficient) = 0, suggesting a downward trend. The overall P-value is 0.002 (*P* for overall = 0.002), which strongly indicates that time has a significant impact on SpO_2_. The non-linear *P*-value of 0.143 (P for nonlinear = 0.143) suggests that the data trend is close to a linear change, with no obvious curve characteristics (Fig. 3M ~ P). The overall relationship between SpO ₂ and protection time shows an inverted "L" shape. These relationships remain even after adjusting for covariates. After adjusting for covariates, pulse pressure exhibits a turning point at 200 min, indicating that, compared to the initial time point at 50 min, at 200 min, the red solid line falls below the gray line with β (regression coefficient) = 0, indicating a downward trend. The overall p-value of 0.008 (*P* for overall = 0.008) strongly suggests that time has a significant effect on pulse pressure; the nonlinear *p*-value of 0.891 (*P* for nonlinear = 0.891) indicates that the data trend is close to linear change, with no obvious curve characteristics (Fig. 3A ~ D). RCS analysis identified a critical inflection point at 200 min of protective exposure. Beyond this threshold duration, hypobaric chamber participants exhibited pronounced physiological alterations, including a precipitous decline in SpO₂, progressive narrowing of pulse pressure, and elevated fatigue perception. These findings collectively indicate the onset of hypoxic stress and concomitant deterioration in work performance efficiency.

### K-M survival curve

**Fig. 4:**
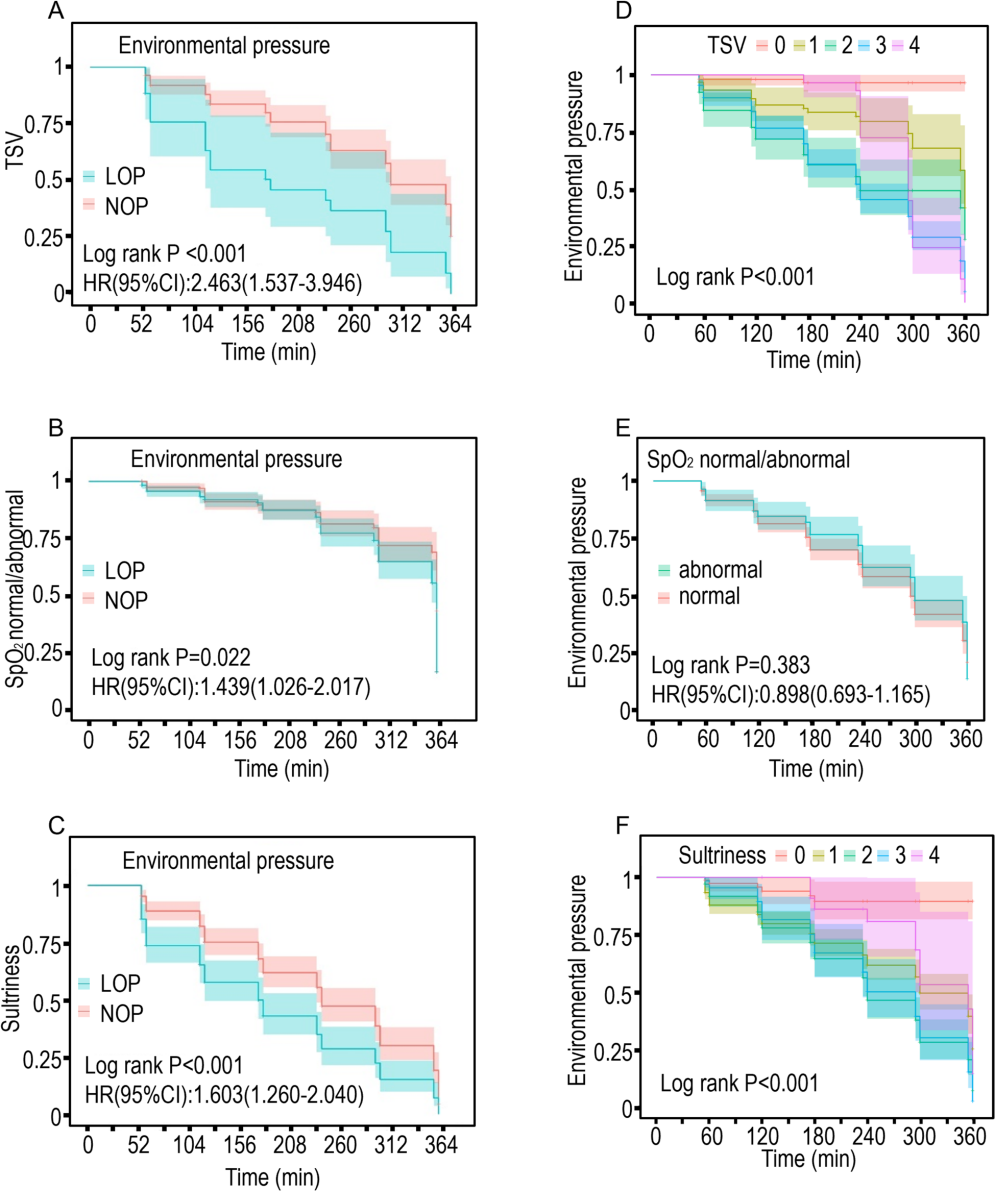
K-M analyses for TSV、SpO_2_ normal/abnormal、Sultriness among the two groups. The solid lines in the figure represents the probability value, and the shaded regions represents the 95% CI. **A** Thermal Sensation Vote; **B** Probability of normal/abnormal of SpO_2_; **C** Sultriness score value; **D** Comparison of TSV probabilities under different protection times; **E** Comparison of Probability of SpO_2_ normal/abnormal under different protection times; **F** Comparison of Probability of Sultriness score value under different protection times

We utilized K-M survival analysis curves to compare differences in TSV, SpO_2_ levels, and Sultriness between participants in the NOP and LOP groups (Fig. 4A ~ C). The results showed significant differences in TSV (HR = 2.463, 95 CI 1.537–3.946, log-rank *P* < 0.001), Sultriness (HR = 1.603, 95 CI 1.260–2.04, log-rank *P* < 0.001), and the probability of normal SpO_2_ levels between the two groups (HR = 1.439, 95 CI 1.026–2.017, log-rank *P* = 0.022). Comparative analysis revealed significant between-group differences in thermal perception and physiological responses. Participants in the LOP environment demonstrated a 2.463-fold greater likelihood of reporting elevated TSV scores compared to the NOP group (Fig. 4A). The temporal progression of thermal discomfort showed marked acceleration under hypobaric conditions, with 50% of LOP participants reaching equivalent thermal sensation levels by 180 min, requiring 300 min in normobaric environments. After 300 min of exposure, 75% of LOP participants reported TSV scores ≥ 4. The probability of SpO_2_ being outside the normal range in the LOP group was 1.439 times that in the normal pressure group (Fig. 4B). The time it takes for 75% of the participants in the LOP group to reach a SpO₂ level out of the normal range (around 230 min) is lower than that of the NOP group (around 312 min). Sultriness perception followed similar patterns, with LOP participants reporting 1.603-fold greater discomfort than NOP counterparts (Fig. 4C). The 50% prevalence threshold for equivalent sultriness levels occurred at 180 min (LOP) versus 250 min (NOP), with 75% of LOP participants scoring ≥ 3 by 300 min of exposure duration. K-M survival analysis curves were also used to compare the incidence of different TSV and sultriness score values, as well as the probability of participants' SpO_2_ being normal or not (Fig. 4D-F). There were significant differences between different score values for both metrics (log-rank *P* < 0.001), while no significant difference was observed between normal and abnormal SpO_2_ levels (log-rank *P* = 0.383). Within 180 min, participants had a higher probability of selecting TSV score value 2 than of selecting TSV scores 3 and 4, with the highest probability of selecting TSV score value 3 at 240 min of protection time, and the highest probability of selecting TSV score value 4 at 300 min (Fig. 4D). For the Sultriness score, the probability of a Sultriness score of 1 for participants within the first 120 min is the highest, and the probability of a Sultriness score of 2 is consistently higher than that of the other three groups (Fig. 4F). SpO_2_ levels were influenced by stress, with an equal probability of normal and abnormal levels within the first 120 min. During the subsequent protection period, the probability of abnormality was greater than normal, but it did not seriously affect the participant's life and health (Fig. 4E). K-M survival analysis demonstrated a significantly greater probability of SpO₂ deviation from normal ranges among participants in hypobaric conditions. Concurrently, these participants exhibited elevated scores on TSV, sultriness perception, and RPE scales, indicating compounded physiological strain manifesting as intensified thermal discomfort and fatigue perception.

### Time series analysis

**Fig. 5:**
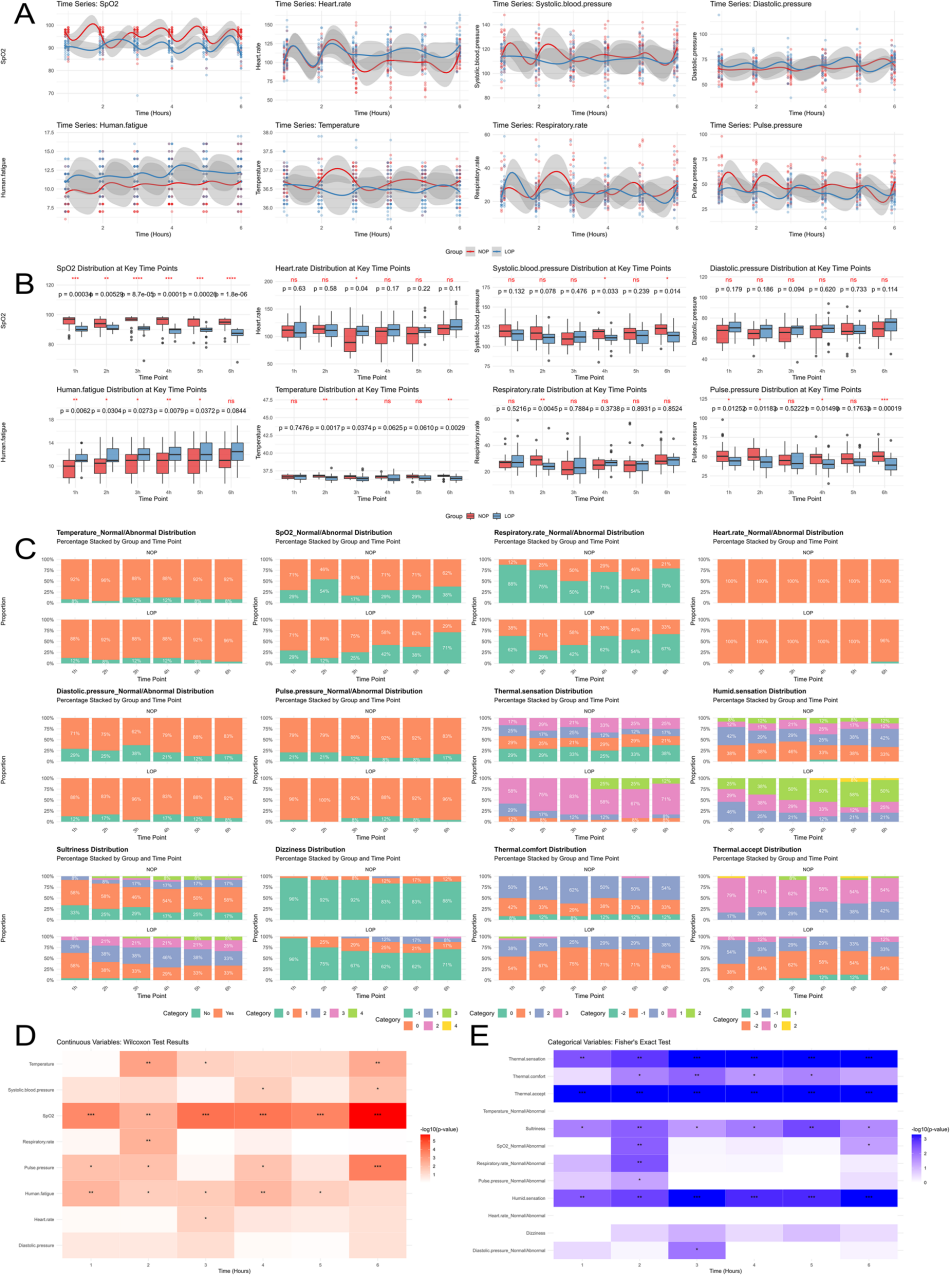
Time series visualization. **A** Visualization of continuous variables. The horizontal axis represents time points from left to right in ascending order, and the vertical axis represents values from bottom to top in ascending order. Red indicates NOP, and blue indicates LOP. **B** Box plot visualization of statistical results for continuous variables. The horizontal axis represents time points from left to right in ascending order, and the vertical axis represents numerical values from bottom to top in ascending order. Red indicates NOP, and blue indicates LOP. **C** Visualization of categorical variables. The horizontal axis is arranged from left to right in ascending order of time points, and the vertical axis shows the proportion of each subgroup of the categorical variable. Temperature_Normal/Abnormal, SpO2_Normal/Abnormal, Respiratory rate_Normal/Abnormal, Heart rate_Normal/Abnormal, Diastolic pressure_Normal/Abnormal, Pulse pressure_Normal/Abnormal share the same legend: green indicates abnormal/no, and orange indicates normal/yes. For other variables, which quantify subjective sensations numerically, the legend follows this order: dark green < orange < blue < pink < light green < yellow. **D** Visualization of statistical test results for continuous variables. The x-axis represents time points from left to right in ascending order, the y-axis represents the variable, and colors range from light to dark corresponding to -log10(*p* value) values from small to large. *P*-values < 0.001 are marked as ***, *P*-values < 0.01 are marked as **, and *P*-values < 0.05 are marked as *. **E** Visualization of statistical test results for categorical variables. The horizontal axis is arranged from left to right in ascending order of time points, the vertical axis represents the variable, and the colors range from light to dark corresponding to the values of -log10(*p* value) in ascending order. *P*-values < 0.001 are marked as ***, *P*-values < 0.01 are marked as **, and *P*-values < 0.05 are marked as *

The time-varying trends of the 8 continuous variables are complex, with no stable trend of NOP being consistently higher or lower than LOP, except for Human fatigue, where LOP has consistently been higher than NOP since the 1-h point; For SpO_2_, NOP remained higher than LOP before 2 h, but after approximately 2.5 h, LOP consistently exceeded NOP; for heart rate, there was no significant difference between the two groups before 2.75 h, but after 2.75 h, the LOP group consistently exceeded the NOP group; no significant patterns of change were observed for the remaining variables (Fig. 5A). The box plot visualization results for continuous variables showed that SpO2 exhibited significantly higher NOP than LOP at all six measurement time points (*P* < 0.05); heart rate only showed significantly higher LOP than NOP at the 3-h time point; SBP only showed significantly higher NOP than LOP at the 4- and 6-h time points; DBP showed no significant differences at any measurement time point; Human fatigue showed no significant difference only at the 6-h time point, with LOP significantly higher than NOP at all other time points; temperature showed NOP significantly higher than LOP only at the 2-, 3-, and 6-h time points; respiratory rate showed NOP significantly higher than LOP only at the 2-h time point; and pulse pressure showed NOP significantly higher than LOP only at the 1-, 2-, 4-, and 6-h time points (Fig. 5B). The composition ratios of categorical variables at the 6 time points are presented using stacked bar charts in Fig. 5C. The statistical test results for all continuous categorical variables at the 6 time points are summarized in Fig. 5D, showing that 2 h was the time point with the most differences (5 variables out of a total of 8 variables). The statistical test results for all categorical variables at the 6 time points are summarized in Fig. 5E. It can be observed that the 2-h time point had the most variables showing differences (8 variables, with a total of 12 variables).

## Discussion

Comparative analysis revealed significant physiological alterations in the LOP group relative to NOP controls. Participants exposed to hypobaric conditions demonstrated clinically relevant reductions in SpO₂ and pulse pressure, concomitant with elevated heart rate and systolic blood pressure. Subjective assessments showed pronounced thermal discomfort, with significantly higher scores for thermal sensation, sultriness, and humidity sensation, alongside diminished thermal comfort and acceptability ratings. These findings collectively indicate that hypobaric exposure exacerbates physiological strain and thermal discomfort during prolonged PPE use. According to the K-M curve, the probability of SpO_2_ abnormality in the LOP group is 1.439 times that of the NOP group (HR 95% CI = 1.439 (1.026—2.017), Log-rank *P* = 0.022) (Fig. 4B). The probability of selecting a high TSV score was 2.463 times higher in the NOP group (HR 95% CI = 2.463 (1.537–3.946), Log-rank *P* < 0.001) (Fig. 4A). The probability of selecting a high sultriness score was 1.603 times higher in the LOP group than in the NOP group (HR 95% CI = 1.603 (1.260–2.040), Log-rank *P* < 0.001) (Fig. 4C). This indicates that, compared to NOP, LOP indeed made subjects more likely to experience discomfort during the 6-h experimental period. Among the continuous variables, at 120 min, it is the moment with the most different variables (*n* = 5, out of a total of 8 continuous variables) (Fig. 5A). Among the categorical variables, at 120 min, it is also the moment with the most different variables (*n* = 8, out of a total of 12 categorical variables) (Fig. 5B). Therefore, the basic statistical tests suggest that the optimal protection time is 120 min. The RCS analysis of NOP is shown in Fig. 2, while the RCS analysis of LOP is shown in Fig. 3. In the results of the RCS curves, the red solid line represents the trend line, and the shaded area indicates the confidence curve. The intersection of the trend line with the horizontal line indicates a critical point, that is, the effect changes before and after this coordinate. Therefore, by examining the results in Fig. 2/3, it is observed that both NOP and LOP, regardless of whether covariates are adjusted, show a turning point at 200 min. That is, when the protection time exceeds 200 min, both LOP and NOP subjects exhibit a gradual decrease in body temperature (Fig. 2I ~ 2L/Fig. 3I ~ 3L) and pulse pressure (Fig. 2A ~ 2D/Fig. 3A ~ 3D). However, in the NOP group, pulse pressure can maintain slight fluctuations between 200 and 250 min (Fig. 2A ~ 2D), and SpO_2_ can maintain slight fluctuations between 125 and 275 min (Fig. 2M ~ 2P). Nevertheless, the changes in pulse pressure (*P* for overall = 0.297) and SpO_2_ (P for overall = 0.840) over the protection time in the NOP group are not significant. However, in the LOP group, the Rating of Perceived Exertion (RPE) (Fig. 3E ~ 3H) increased after 200 min, while SpO_2_ (Fig. 3M ~ 3P) showed a direct downward trend after 200 min, indicating that the maximum tolerable time for LOP is 200 min. Therefore, RCS indicates that the maximum protective time for the LOP group is 200 min. These temporal patterns establish 120 ~ 200 min as the maximum recommended exposure duration for medical personnel operating in hypobaric environments while wearing secondary PPE.

Medical protective clothing serves as a critical barrier against microbial transmission while ensuring healthcare worker safety. However, the impermeable materials creating this protective barrier simultaneously generate a thermally stressful microenvironment. This thermal burden becomes particularly pronounced during aeromedical evacuations of infectious disease patients, where medical personnel must contend with compounded environmental stressors, including hypobaric conditions and elevated ambient temperatures. The 2014 Ebola virus disease outbreak in West Africa demonstrated these challenges, with the United States successfully evacuating two infected patients from Liberia via modified Gulfstream III aircraft. This specialized medical transport, capable of 928 km/h cruising speeds, completed the transatlantic evacuation to Emory University Hospital within approximately 4 h [39]. Similarly, Chinese aeromedical operations typically employ fixed-wing aircraft for patient transfers, with maximum documented transport durations reaching 5 h over distances of 2,650 km [39, 40]. These extended flight durations necessitate prolonged exposure of medical staff to the combined physiological stresses of cabin pressure alterations and thermal microenvironment constraints within protective equipment. Empirical evidence demonstrates that elevated ambient temperatures induce significant physiological alterations, including increased core body temperature, elevated heart rate, and enhanced fluid loss, while concurrently impairing task performance relative to thermoneutral conditions [14]. Comparable physiological strain emerges during strenuous activity in thermally challenging environments, manifesting as reduced tolerance duration, diminished cognitive function (including attention, reaction time, and verbal memory), and heightened subjective fatigue perception [41–43]. Low-pressure environments can lead to a decrease in heart rate deceleration force, work efficiency, working memory, and attention in healthy individuals, while increasing the risk of elevated hemoglobin levels [23, 44, 45]. These effects are exacerbated during physical exertion, potentially compromising myocardial oxygen delivery [46]. While existing research has extensively characterized human physiological and psychological responses to either thermal stress or hypobaric hypoxia independently, the synergistic effects of combined thermal and hypobaric stressors remain poorly understood. To address this knowledge gap, we conducted a controlled investigation monitoring physiological parameters and subjective responses in 24 participants during 6-h exposures to both NOP and LOP conditions while wearing personal protective equipment and performing standardized activities.

Acute exposure to hypobaric environments induces multifaceted physiological disturbances primarily mediated through hypoxic biochemical pathways. These pathophysiological responses encompass neurological manifestations (headache, dizziness) [47, 48], cardiovascular alterations (blood pressure instability, myocardial ischemia) [19], and thermoregulatory dysfunction (impaired heat dissipation, metabolic dysregulation) [49]. Our experimental data demonstrate that LOP participants exhibited significantly reduced pulse pressure, core temperature, and SpO₂ compared to NOP controls. Hypoxic stimulation of the sympathetic nervous system [50, 51], triggers compensatory cardiovascular responses, including elevated heart rate and cardiac output. This hemodynamic adaptation becomes particularly detrimental in hyperthermic conditions, where cutaneous vasodilation imposes additional cardiac workload [52]. Empirical evidence indicates that combined exposure to 3,000 m altitude and 35 °C ambient temperature elevates resting heart rate by 15–20 bpm and increases myocardial oxygen demand by 30% [53]. The hypoxic stress response further promotes catecholamine release [54], inducing visceral and renal vasoconstriction that elevates diastolic pressure and partially counteracts thermal vasodilation [55]. Concurrently, profuse sweating precipitates hypovolemia, diminishing cardiac output, and exacerbating systolic pressure reduction [56]. This mechanistic cascade explains the progressive pulse pressure decline observed in our hypobaric cohort. Thermoregulatory measurements revealed parallel declines in core temperature with prolonged exposure, albeit with limited variability. Under normal conditions, when participants work in protective clothing for 6 h, their body temperature should show an upward trend. However, in the hypobaric chamber, the combined effects of high temperature, low pressure, and hypoxia may cause evaporative and convective heat dissipation to far exceed the body's heat production capacity [57]. Sweat evaporates more easily, taking away a large amount of heat [58]. The resultant hypovolemia from inadequate fluid replacement impaired circulatory heat redistribution from core to periphery, manifesting as paradoxical core temperature reduction.

SpO₂ serves as a clinically significant biomarker for evaluating tissue oxygenation status, reflecting the oxygen-binding capacity of hemoglobin. This noninvasive measurement provides a quantitative assessment of hypoxic stress, cardiovascular workload, and pulmonary function. SpO₂ measurements demonstrate sensitivity to both physiological activity levels and environmental conditions, particularly atmospheric pressure variations. Clinical observations indicate that when SpO₂ levels decline below 85%, neurocognitive impairment becomes evident, manifesting as diminished concentration capacity and compromised fine motor coordination [59]. The SpO_2_ levels of participants in both groups in this study changed minimally in the first 200 min and began to decline after this period, with a higher rate of decline in the LOP group compared to the NOP group (Figs. 2 and 3). At the end of the trial, participants in the NOP group had an average SpO_2_ of 94.04%, while those in the LOP group had an average SpO_2_ of 86.48%. K-M survival curves showed that the probability of maintaining normal SpO_2_ for participants in the LOP group was lower than that in the NOP group when the duration of protection exceeded 200 min (Fig. 4). The physiological response to acute hypobaric hypoxia involves compensatory mechanisms to maintain tissue oxygenation. During initial exposure, respiratory frequency and tidal volume increase to elevate alveolar oxygen partial pressure, while tachycardia develops to sustain peripheral oxygen delivery [60]. Concurrent thermal stress induces cutaneous vasodilation to facilitate heat dissipation, while sympathetic-mediated visceral vasoconstriction redistributes blood flow to prioritize cerebral and myocardial oxygenation, thereby delaying initial SpO₂ decline [51, 61, 62]. On the other hand, in the early stages of dehydration, blood concentration leads to a relative increase in hemoglobin concentration, and the oxygen-carrying capacity is not significantly impaired, thus stabilizing SpO₂ [63, 64]. As the protection time extends, participants who have not eaten or drunk experience continuous dehydration, leading to a reduction in blood volume, decreased preload on the heart, and a drop in stroke volume. Meanwhile, high temperatures increase the demand for blood flow to the skin, and the cardiac output cannot meet the dual load, resulting in a collapse of tissue oxygen delivery [65, 66]. Studies have shown that in hypoxic environments, deoxygenated hemoglobin in skeletal muscles rises sharply after 200 min, reflecting an impairment in oxygen utilization. Concurrently, increased anaerobic glycolysis leads to elevated blood lactate concentrations, with blood pH levels dropping below 7.2. This inhibits the oxygen-binding capacity of hemoglobin, creating a vicious cycle [67–69]. Similarly, Simone Porcelli et al. studied the respiratory and blood responses to hypoxia in 13 healthy participants exposed to low-pressure hypoxic environments and found that SpO_2_ remained below pre-exposure levels without showing any upward trend [70]. The decrease in SpO_2_ with increasing altitude was also observed in expedition members by SY Zhao [71]. Our research found that wearing protective clothing for extended periods can lead to participants being in a state of hypoxia, and the low-pressure environment exacerbates this effect, which is consistent with most existing studies.

TSV and sultriness represent subjective evaluations of ambient thermal conditions and personal thermoregulatory status. K-M analysis demonstrated a time-dependent escalation in both TSV and sultriness scores with prolonged exposure duration. Comparative analysis revealed that participants in the LOP environment exhibited a 2.463-fold higher likelihood of reporting elevated TSV scores compared to the NOP group (Fig. 4D). Temporal progression analysis showed that the median time to reach equivalent thermal sensation levels was 180 min under hypobaric conditions versus 300 min in normobaric environments. At 300 min of exposure, 75% of LOP participants reported TSV scores ≥ 4, indicating substantial thermal discomfort (Fig. 4D). Similarly, sultriness perception was significantly amplified in hypobaric conditions, with LOP participants demonstrating 1.603-fold greater discomfort than NOP controls (Fig. 4F). The temporal threshold for 50% of participants to report comparable sultriness levels was reduced by 28% in hypobaric environments (180 min) relative to normobaric conditions (250 min). By 300 min of exposure duration, 75% of LOP participants reported sultriness scores ≥ 3, confirming progressive environmental discomfort (Fig. 4F). Statistical analysis confirmed significant between-group differences in both thermal perception parameters (*p* < 0.01). TSV and Sultriness scores are based on their surroundings and can be affected by the exchange of heat and moisture between the body and the external environment. According to the different exchange mechanisms, the heat exchange between the human body and the surrounding environment typically involves four mechanisms: conduction, convection, radiation, and evaporation. In low-pressure environments, air density decreases due to lower atmospheric pressure, which weakens convective heat dissipation while enhancing evaporative heat dissipation [57]. Additionally, the clothing worn can affect the convective heat exchange between the body and the surrounding air, and protective clothing made of special materials can also reduce the evaporation heat dissipation rate [72]. The TSV and Sultriness score values increase when participants are subjected to both protective clothing and low-pressure environments. Our findings confirm that as the duration of protection increases, participants' subjective feelings change, with a gradual rise in TSV and Sultriness becoming more pronounced, making them more prone to uncomfortable sensations such as stuffiness, dizziness, and fatigue.

Human fatigue is characterized by a sense of physical discomfort and a state of physical and mental overload. Poor thermal comfort can readily lead to fatigue. When the body experiences fatigue, behavioral ability begins to decline, and attention cannot be focused [73]. The Mann–Whitney test revealed a significant difference in fatigue levels between the two groups, with an L-shaped relationship between fatigue and protection time in the LOP group, reaching a minimum around 200 min. In the NOP group, the fatigue level initially increased with protection time, then decreased, and increased again, but the changes were relatively small. Hypobaric hypoxia elicits sympathetic nervous system activation and stress responses that heighten thermal discomfort perception, amplifying subjective heat sensation at equivalent ambient temperatures. Walsh et al. demonstrated that acute aerobic exercise at 4,240 m altitude significantly impaired cognitive performance, including work efficiency, working memory, and attention span, compared to 1,400 m conditions [23]. Complementing these findings, Ochi et al. documented progressive altitude-dependent work capacity reductions across 0–5,000 m elevations in 21 participants [21]. Thermoregulatory pathways involving pontine parabrachial nuclei and hypothalamic neurons become activated under thermal stress, potentially inducing central fatigue and cognitive impairment during prolonged exposure [74]. This mechanism aligns with our observed RPE elevation in later experimental phases. Hypobaric conditions additionally stimulate hypothalamic–pituitary–adrenal (HPA) axis activation, triggering catecholamine and cortisol release that transiently enhances cardiovascular performance (increased heart rate, alertness, and muscle contractility) while attenuating fatigue perception [75]. This neuroendocrine response explains the initial RPE reduction in hypobaric-exposed participants. Progressive dehydration induces hemoconcentration and electrolyte imbalances, impairing cardiovascular function (reduced stroke volume) and neuromuscular transmission [76, 77]. Compensatory tachycardia develops to maintain cutaneous perfusion for thermoregulation, while sustained cardiac strain precipitates cumulative "latent fatigue" manifesting as delayed RPE escalation [78]. Extended fasting during protocols depletes glycogen reserves, compromising ATP synthesis and muscular performance [79, 80]. Critical physiological deterioration occurs when SpO₂ declines precipitously, as hypobaric hypoxia diminishes cerebral perfusion and prefrontal cortex activity, impairing attention and inducing cognitive fatigue. Concurrent neurotransmitter dysregulation (e.g., serotonin) exacerbates somnolence and further elevates RPE [81]. Subjective indicators are easily influenced by individual differences. For example, individuals with experience in high-temperature environments or those who have worn protective clothing for extended periods have a more adaptive thermoregulatory system, and taller individuals generate more heat [82]. Therefore, to minimize the interference of confounding factors, we imposed restrictions on the participants' height, age, training background, BMI, and weight.

This study investigated the changes in physiological indicators and subjective feelings under prolonged exposure to a low-pressure environment while wearing protective clothing. However, there are some limitations to consider: 1) The failure to have participants complete the questionnaire at the initial moment (time 0) resulted in incomplete baseline data, which also affected the completeness of the RCS curves; 2) The number of participants participating in the experiment was small, and the results may be biased; 3) The participants underwent human thermal physiology experiments in a low-pressure oxygen chamber. Two weeks later, the same participant underwent human thermal physiology experiments under normal atmospheric pressure following the same procedure. Although there was a two-week washout period, the low-pressure oxygen chamber might still have a slight impact on the subjects.

## Conclusions

Our study demonstrated that barometric pressure significantly altered participants' SpO_2_, DP, SBP, temperature, HR, and pulse pressure. As the duration of protection increased, participants' TSV, Sultriness, and RPE scores gradually increased. We found that four hours is the optimal working duration that does not adversely affect the efficiency of medical staff.

## Supplementary Information

Supplementary Material 1. Questionnaire on Thermal Comfort of medical personnel in protective clothing

Supplementary Material 2. Informed Consent Statement

## Data Availability

All data generated or analyzed during this study are included in this Published article and its supplementary information files.

## Abbreviations

LOP: Low oxygen pressure
NOP: Normal oxygen perssure
CPR: Cardio pulmonary resuscitation
RCS: Restricted cubic spline
K-M: Kaplan Meier
TSV: Thermal sensation vote
RPE: Rate of perceived exertion
HR: Heat rate
SpO_2_: Oxygen saturation
BMI: Body Mass Index
SBP: Systolic blood pressure
DP: Diastolic pressure
ECG: Electrocardiogram monitor
TCV: Thermal Comfort Vote
TAV: Thermal Acceptance Vote
HSV: Humid Sensation Vote
