# Supplementary material for: Changes in physiological parameters and thermal comfort when wearing protective clothing in long-range aeromedical evacuation: a prospective, non-blinded, two-stage crossover self-controlled study
Source: J Physiol Anthropol. 2025 Nov 7;44:28. doi: 10.1186/s40101-025-00411-9 (PMC12595711; doi:10.1186/s40101-025-00411-9)

Supplementary material 1

Number： Name： Gender： Height/Weight： m/ kg BMI： kg/m^2^ Age： Date： Time： hour min

1. Which part of your body do you feel sweating the most at this time? (Multiple choices are available, please arrange them in order of 1, 2, 3, 4..., if none are available, do not select)

Scalp ( ) Face ( ) Neck ( ) Hands ( ) Forearms ( ) Back ( Upper ) Arms ( ) Chest ( ) Back ( ) Abdomen ( ) Hips ( ) Thighs ( ) Calfs ( ) Soles of Feet ( )

2. Do you feel difficulty breathing/feeling suffocated? No (); Somewhat (); Very strong ()

3. At this moment, what feeling can't you tolerate the most: heat; Wet ()

4. TSV

| TSV  level | Chilly | Cold | Cool | Slightly cool | Moderate | Slightly warm | Warm | Heat | Hot |
| --- | --- | --- | --- | --- | --- | --- | --- | --- | --- |
|  | -4 | -3 | -2 | -1 | 0 | 1 | 2 | 3 | 4 |

Moderate means neither cold nor hot; Slightly warm indicates feeling a little warm just now; Warm indicates a feeling of warmth, but without sweating; Heat indicates the onset of sweating; Hot indicates continuous sweating and a strong feeling of stuffiness.

5. HSV

| HSV level | Very dry | Extremely dry | Dry | Slightly dry | Moderate | Slightly moist | Moist | Extremely moist | Very moist |
| --- | --- | --- | --- | --- | --- | --- | --- | --- | --- |
|  | -4 | -3 | -2 | -1 | 0 | 1 | 2 | 3 | 4 |

HSV: Humidity Sensation Vote; Moderate means that the body's skin is neither dry nor moist; Slightly moist indicates that the skin is starting to feel moist, but there is no sweating; Extremely moist indicates that the body is starting to sweat and there are noticeable beads of sweat on the skin; Very moist indicates continuous heavy sweating and moist skin.

6. TCV

| TCV level | Very discomfort | discomfort | Slightly discomfort | Moderate | Slightly discomfort | discomfort | Very discomfort |
| --- | --- | --- | --- | --- | --- | --- | --- |
|  | -3 | -2 | -1 | 0 | 1 | 2 | 3 |

Moderate means neither hot nor sweaty; Slightly discomfort indicates a feeling of warmth and slight sweating; Discomfort indicates the onset of stuffiness and sweating; Very discomfort indicates continuous heavy sweating and difficulty breathing.

7. TAV

| TAV level | Completely unacceptable | Slightly unacceptable | Moderate | Slightly acceptable | Completely acceptable |
| --- | --- | --- | --- | --- | --- |
|  | -2 | -1 | 0 | 1 | 2 |

TAV: Thermal Acceptability Vote; Moderate indicates that there are no abnormal sensations in the body; Slightly unacceptable indicates that the body is starting to feel uncomfortable; Completely unacceptable with the expression of difficulty breathing, dizziness, and suffocation, and immediately stop the experiment.

8. RPE

The subjective human fatigue evaluation index uses the Borg 15 level RPE subjective sensation scale for fatigue level. The human fatigue level ranges from 6 to 20, with higher values indicating a stronger sense of fatigue. Grade 6 is very easy, no fatigue, the body feels full of spirit; grade 13 means a little effort, the body appears fatigue, energy can not be concentrated; grade 17 means very effort, fatigue is strong, the body appears to be loaded movement; grade 20 means very effort, almost reached the limit of the human body, the body can not withstand the load, continue to exercise will be a threat to the safety of life.


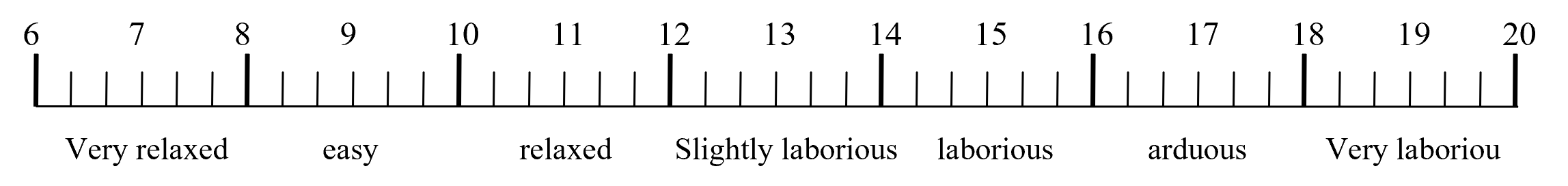

Supplement: Supplementary file 1 — Supplementary Material 1. Questionnaire on Thermal Comfort of medical personnel in protective clothing [file 40101_2025_411_MOESM1_ESM.docx]
