# Supplementary material for: Changes in physiological parameters and thermal comfort when wearing protective clothing in long-range aeromedical evacuation: a prospective, non-blinded, two-stage crossover self-controlled study
Source: J Physiol Anthropol. 2025 Nov 7;44:28. doi: 10.1186/s40101-025-00411-9 (PMC12595711; doi:10.1186/s40101-025-00411-9)
Supplement: Supplementary file 2 — Supplementary Material 2. Informed Consent Statement [file 40101_2025_411_MOESM2_ESM.docx]

Supplementary material 2

Dear participant:

We invite you to participate in the research project of the 2022 Science and Technology Assistance Program of the Air Force Characteristic Medical Center. This study will be conducted at the Air Force Characteristic Medical Center, and 24 participants will voluntarily participate. The study has been reviewed and approved by the Ethics Committee of the Air Force Characteristic Medical Center of the People's Liberation Army.

Why conduct this research?

Air medical evacuation is a method of using fixed wing aircraft, helicopters, and other transportation tools to quickly and promptly transport wounded and sick patients to specialized treatment institutions for professional and definitive treatment. In the process of transporting high-risk infectious disease patients by air, medical personnel need to wear protective clothing to carry out rescue activities for the injured and sick. Due to changes in altitude, air pressure, acceleration, temperature during the flight, as well as factors such as limited space, noise, and vibration on the aircraft, not only does the rescue operation in air transportation differ from traditional rescue modes, but it also puts higher demands on the physical fitness of medical personnel.

Medical protective clothing provides a barrier and protection for medical personnel to come into contact with potentially infectious patient blood, body fluids, secretions, particulate matter in the air, etc. during work, and is an important guarantee for maintaining the life and health of medical personnel. In order to effectively prevent harmful microorganisms, protective clothing is made of various composite materials and must be "airtight". When carrying out the task of airlifting wounded and sick, medical personnel are exposed to a humid, low oxygen, and low-pressure microenvironment under protective conditions, which can easily cause chest tightness, poor breathing, and fatigue. In addition, there are challenges such as inability to eat, difficulty excreting, high intensity, and long working hours, which pose a risk of inducing diseases and leading to medical accidents. Studies have shown that in hot and humid environments, when the ambient temperature is above 30°C, even if the human body is in a quiet state, the body temperature rises and the heart beats faster. Therefore, in order to protect the physical and mental health of medical staff, it is necessary to conduct research on the human body under protective conditions, analyze the impact of working time and intensity on the physiological indicators of medical staff under medical protective conditions.

Who can participate in the research?

The inclusion criteria for the study population are: (1) healthy adults aged 20-30 years old; (2) No history of chronic illness; (3) No aviation contraindications (patients with respiratory diseases, severe anemia, cerebrovascular diseases, cardiovascular diseases, psychiatric patients).

Who is not suitable to participate in the research?

Exclusion criteria: (1) Recent ophthalmic surgery (cataract surgery), mandibular fixation surgery, abdominal surgery; (2) Recently suffering from acute gastrointestinal diseases and severe colds; (3) Suffering from airsickness.

What do I need to do if I participate in the research?

If you are willing to participate in this study, you will accept the following three experimental plans:

**1. Normal atmospheric pressure without protective clothing**

1.1 Set the temperature and humidity of the testing site to ensure a stable testing environment.

1.2 The subjects rest outside the experimental site for 15 minutes, empty their bladder and stool, and stabilize their physiological state.

1.3 Wear an electrocardiogram monitor for the subjects, turn on the instrument, and monitor physiological indicators.

1.4 The subject sits quietly for 50 minutes, steps at a frequency of 75 times per minute (70-80 steps) for 5 minutes, rests for 3 minutes, and perform 5 sets of CPR. Fill out the questionnaire after stepping and CPR. Cycle 2 groups, fasting and no water throughout the entire process.

1.5 If you feel unwell during the research process and confirm that you have reached your limit, we will stop the experiment.

**2. Protective clothing under normal atmospheric pressure**

1.1 Set the temperature and humidity of the testing site to ensure a stable testing environment.

1.2 The subjects rest outside the experimental site for 15 minutes, empty their bladder and stool, and stabilize their physiological state.

1.3 Wear an electrocardiogram monitor for the subjects, wear protective equipment according to the second level protection requirements, turn on the instrument, and monitor physiological indicators.

1.4 The subject sits quietly for 50 minutes, steps at a frequency of 75 times per minute (70-80 steps) for 5 minutes, rests for 3 minutes, and perform 5 sets of CPR. Fill out the questionnaire after stepping and CPR. Cycle 6 groups, fasting and no water throughout the entire process.

1.5 If you feel unwell during the research process and confirm that you have reached your limit, we will stop the experiment.

**3. Protective clothing for low-pressure oxygen compartments**

1.1 Adjust the simulation parameters of the low-pressure cabin to simulate the internal environment of the cabin of a medical ambulance aircraft at a flight altitude of 2500m above ground. The test is conducted after the cabin environment is stabilized.

1.2 The subjects rest outside the experimental site for 15 minutes, empty their bladder and stool, and stabilize their physiological state.

1.3 The subject was fitted with a cardiac monitor, dressed in protective gear in accordance with the requirements for secondary protection, entered a low-pressure oxygen chamber, turned on the instrument, and had physiological indicators monitored.

1.4 The subjects sit quietly for 50 minutes, step at a frequency of 75 times per minute (70-80 steps) for 5 minutes, rest for 3 minutes, and perform 5 sets of CPR. Fill out the questionnaire after stepping and CPR. Cycle for 6 groups, fasting and water restriction throughout the entire process.

1.5 If you feel unwell during the research process and confirm that you have reached your limit, we will stop the experiment.

What are the risks of participating in research?

Medical protective clothing has isolation of germs, harmful ultrafine dust, acidic and alkaline solutions, electromagnetic radiation, etc., can guarantee the safety of witnesses and keep the environment clean. In order to enhance the protective effect, protective clothing fabrics are usually laminated or specially treated, resulting in thick and heavy and poor breathability and moisture permeability, which is not conducive to perspiration and heat exhaustion when worn for a long period of time. As a result, you may experience symptoms such as breathlessness, chest tightness, and dizziness during the study. In addition, the low-pressure environment may cause adverse reactions such as altitude sickness (chest tightness, shortness of breath, panic, headache, dizziness, inability to walk, etc.), cerebral hypoxia, and pulmonary hypertension. During the study, we will prepare oxygen tanks and medications such as cytarabine sodium, piracetam, digoxin, warfarin, etc. in advance and arrange two healthcare workers to stand by to ensure basic first-aid protection, especially the emergency treatment of hypoxia and dehydration. In case of research-related damages, subsequent treatment costs and compensation will be our responsibility.

What are the benefits of participating in the study?

Through this study, we can obtain experimental data on the changes of physiological indicators of the human body over time under the conditions of protective clothing, which will provide a basis for the maximum time that medical personnel can withstand medical activities in protective clothing on board the aircraft.

Are there costs associated with participating in the study?

To compensate you for the inconvenience that your participation in this study may cause you, we will pay you a lump sum of $500 for your labor by bank card transfer after you have completed two trials. The test site, test materials and equipment required during this study will be provided by us at no cost to you.

Is personal information confidential?

Your personal data will be used only for this study and will be kept at the Center, and the investigators, research authorities, and ethics committees will be granted access to your data records. We will make every effort to protect your privacy to the extent permitted by law.

Do I have to participate in the study?

Participation in this study is completely voluntary, and you may refuse to participate in the study or withdraw from the study at any time during the study, which will not affect your health. If you do not participate in this study, or if you withdraw from the study halfway through, please contact your doctor, and you may be asked to undergo tests that may be beneficial to protect your health.

**Subject Statement:** I have read the above description of this study and fully understand the possible risks and benefits of participating in this study. I volunteer to participate in this study.

**I agree□ or refuse□** Studies other than this one utilized my medical records and pathology specimens.

Subject's signature: 　 Date:＿ ＿ ＿ ＿ year ＿ ＿ month ＿ ＿ day

Subject's mobile phone number:

Signature of legal representative: Date:＿ ＿ ＿ ＿ year ＿ ＿ month ＿ ＿ day

Legal representative's cell phone number: Relationship with subjects:

**Researcher Statement:** I confirm that the details of this study have been explained to the subject, in particular the possible risks and benefits of participating in this study.

Signature of the researcher: Date:＿ ＿ ＿ ＿ year ＿ ＿ month ＿ ＿ day

Researcher's work phone: Cell phone number:

Air Force Specialty Medical Center Ethics Committee Office Contact Tel: 010-66928575
